# Occupational Therapy for Children With DCD and Academic Difficulties: A Pan-Canadian Survey

**DOI:** 10.1177/00084174251359768

**Published:** 2025-07-30

**Authors:** Eliane Dionne, Annette Majnemer, Miriam H. Beauchamp, Marie Brossard-Racine

**Keywords:** Academic activities, Developmental coordination disorder, Occupational therapy, Practices, Survey, Activités scolaires, ergothérapie, pratiques, sondage, trouble développemental de la coordination

## Abstract

**Background.** Children with Developmental Coordination Disorder (DCD) often experience academic challenges. Although children with DCD are frequently referred to Occupational Therapy (OT) to help alleviate some of their motor and functional challenges, the actual practices of OTs regarding academic activities remain underexplored in this group. **Purpose.** This study aimed to describe the nature and extent of Canadian OT practices regarding academic activities in children with DCD. **Method.** A survey was sent to pediatric OTs through national and provincial OT associations and licensing organizations, to gather information on assessment and intervention practices related to core academic activities in children with DCD. **Findings.** A total of 229 OTs completed the survey (170 females, 74%). Occupational Therapies reported assessing or providing intervention for academic activities, most frequently handwriting (96% assessed and 85% intervened), writing (74% and 65%), mathematics (72% and 68%), and reading (66% and 59%). With respect to intervention services, up to 78% of OTs provided direct intervention, compared to 51% for indirect or consultative services. **Conclusion.** Most Canadian pediatric OTs typically assess and provide intervention for handwriting and, to a lesser extent, other academic activities. These practices vary depending on work setting and experience.

## Résumé

**Description.** Les enfants ayant un trouble développemental de la coordination (TDC) présentent souvent des difficultés scolaires. Bien que ces enfants soient fréquemment orientés vers l'ergothérapie pour atténuer leurs défis moteurs et fonctionnels, les pratiques des ergothérapeutes en lien avec les activités scolaires restent peu explorées chez cette population. **But.** Cette étude vise à décrire la nature et l’étendue des pratiques des ergothérapeutes canadiens concernant les activités scolaires des enfants avec un TDC. **Méthodologie.** Un sondage a été envoyé aux ergothérapeutes en pédiatrie via leurs associations et ordres professionnels afin de recueillir des informations sur les pratiques d’évaluation et d’intervention liées aux activités scolaires chez les enfants ayant un TDC. **Résultats.** Au total, 229 ergothérapeutes ont répondu au sondage (170 femmes, 74%). Les ergothérapeutes ont rapporté évaluer ou intervenir principalement sur les activités scolaires suivantes : l’écriture manuelle (96% évaluent et 85% interviennent), les aspects non-moteurs de la rédaction (74% et 65%), les mathématiques (72% et 68%) et la lecture (66% et 59%). En ce qui concerne les services d’intervention, jusqu’à 78% des ergothérapeutes ont indiqué offrir des services d’intervention directs, comparativement à 51% pour les services indirects ou de consultation. **Conclusion.** La majorité des ergothérapeutes pédiatriques canadiens évaluent et interviennent principalement sur l’écriture manuelle, et sur d’autres activités scolaires mais dans une moindre mesure. Ces pratiques varient en fonction du milieu de travail et de l'expérience des ergothérapeutes.

## Introduction

Developmental coordination disorder (DCD) is a chronic condition that impacts motor skills and coordination and affects about five percent of the school-aged population worldwide (Blank et al., 2012). The symptoms of DCD include difficulties with balance, visual-motor integration, agility, and coordination, as well as other symptoms such as poor body awareness and visual perception, all of which can negatively affect participation in daily life to include school-based activities (Blank et al., 2012). As many as 90% of children with DCD experience handwriting or mathematical difficulties, while 40% have a co-occurring specific learning disorder in reading or written expression (Dionne et al., 2022).

Occupational therapists (OTs) play a pivotal role in the rehabilitation of children with DCD, as they are uniquely positioned to assess the functional impacts of their motor difficulties and to deliver targeted and individualized interventions to alleviate these activity limitations. Specifically, OTs have a unique expertise to document and confirm the first two diagnostic criteria of the DSM-5 for DCD which pertain to the identification of significant motor difficulties and their functional impacts during activities of daily living and/or academic productivity (American Psychiatric Association, 2013). While this second criterion explicitly includes academic activities as a potential area of difficulty for children with DCD, it remains unclear whether OTs actively address this area of functioning in their management of children with DCD. While handwriting difficulties are a common reason for referral to OT (Nightingale et al., 2022), OTs can improve participation in all academic activities by modifying or adapting both the tasks and the environment, making these more accessible to children with disabilities, including those with DCD (Strong et al., 2018).

Academic activities, which include writing (consisting of handwriting and the nonmotor aspects of writing such as sentence construction, grammar, spelling, etc.), reading, and mathematics, constitute the bases of scholarly competencies. Children with DCD experience difficulties performing these activities, with handwriting being the most widely recognized difficulty due to its motor-based nature (Huau et al., 2015; Magalhaes et al., 2011; Rosenblum & Livneh-Zirinski, 2008). Nevertheless, a recent systematic review revealed that up to 90% of children with DCD also experience difficulties in mathematics, and that they perform on average one standard deviation below their typically developing peers in nonmotor aspects of writing, reading, and mathematics (Dionne et al., 2022). These mathematical difficulties have been shown to be associated with poor visual-perceptual skills and are likely exacerbated by the motor components and the time constraints included in many school tasks (Dionne et al., 2024). Collectively, experiencing academic difficulties is associated with lower levels of education, socioeconomic status, and quality of life (Cousins & Smyth, 2003; Patton et al., 1997). Therefore, it is crucial for OTs to be fully aware of the impacts of DCD on all academic activities to ensure effective management of all relevant activity limitations and make appropriate referrals to additional professionals when necessary.

An array of approaches is available to OTs to leverage the personal strengths of children with DCD and target their varied challenges, such as task-specific training, cognitive, sensorimotor, functional, perceptual-motor, environmental, sensory integration, and behavioral approaches (Mandich et al., 2001; Withers et al., 2017). The international clinical practice recommendations on the management of difficulties experienced by children with DCD, including for academic challenges, support the use of task- or activity-oriented approaches as these yield better and faster functional performance outcomes in children with DCD than process-oriented approaches (Blank et al., 2019; Blank et al., 2012; Smits-Engelsman et al., 2013; Sugden & Chambers, 2003). For instance, the Cognitive Orientation to Daily Occupational Performance approach (CO-OP), which focuses on verbally generating cognitive strategies to address specific problematics, is such an evidence-based approach and can be used to alleviate academic difficulties (Sangster et al., 2005; Thornton et al., 2016). A survey of OT practices within children with DCD in British Columbia (Canada) reported that in 2014, 90% of pediatric OTs provided intervention for self-care activities, while only 2% of OTs focused on preprinting and handwriting skills (Withers et al., 2017). This suggested that the frequent academic challenges encountered by children with DCD were not systematically addressed by OTs at that time, possibly due to organization of and access to services (Camden et al., 2019). While OT practices may have changed since the publication of this survey, especially considering the increased recognition of evidence on children's academic difficulties in this population, these remain unclear (Dionne et al., 2022). A thorough understanding of the role of Canadian OTs with children with DCD is a key stepping stone toward reinforcing their role as pediatric OTs and supporting the application of best practice.

## Study Objectives

The primary objective of this study was to determine the nature and extent of Canadian OTs’ assessment and intervention practices as related to academic activities in school-aged children with DCD. To identify potential trends in practices, associations between participant or service characteristics and OT practices were investigated as a secondary exploratory objective.

## Method

### Participants

Canadian OTs with an active license at the time of the study (2023) were recruited. They had to be proficient in either French or English with a minimum of one year of pediatric clinical experience and have had at least one client with DCD in their caseload during the previous year. Only participants who filled out at least one of the core sections, Assessment Practices or Intervention Practices, were included in the data analysis.

### Procedures

This cross-sectional study surveyed a convenience sample, applying snowball sampling, of OTs across Canada. The study was conducted in accordance with the Declaration of Helsinki. Scientific and Ethical approval was obtained from the McGill University Health Centre's Pediatric Research Ethics Board prior to recruitment. The survey was made accessible through the online Limesurvey platform (LimesurveyGmbH). The open survey was distributed by email between October 1, 2023, and December 3, 2023, to potential participants through the recognized professional OT colleges, associations, orders, or societies of New Brunswick, Quebec, British Columbia, Prince Edward Island, Ontario, Alberta, Nova Scotia, and Manitoba. The Northwest territories, Yukon, Nunavut, Saskatchewan, and Newfoundland and Labrador were reached through the Canadian OT Association. Additionally, the survey was distributed within the research team's networks, including McGill University, University of British Columbia, Western University, Canchild from McMaster University, the *Dagobert et cie*, Association and targeted social media groups (e.g., Facebook pages for: McGill School of Physical and Occupational Therapy *SPOT*, *Ergothérapie-Quebec*, and *Ergothérapie en milieu scolaire)*. Electronic consent was obtained at the beginning of the survey and prior to filling out any questions and eligibility was confirmed using self-report questions. Cookies were used to prevent repeat participation. After the recruitment phase, 16 participants among those who had opted into the draw were selected to receive a $25 gift card.

### Survey Development

The survey was developed by a panel of OT researchers using the results of a systematic review on academic difficulties in children with DCD (Dionne et al., 2022) and the CHERRIES checklist (Eysenbach, 2004). Then, four OTs, two francophone and two anglophone, each with a minimum of five years of experience in pediatrics, field-tested the survey by evaluating the clarity of each question using a three-point Likert scale (very clear, somewhat clear, unclear). If any item was deemed unclear, the OTs were asked to provide suggestions for improvement. Feedback was reviewed and revised versions of the questions were proposed for reevaluation. Iterative revisions were made until consensus was reached among the participating OTs and the survey developers. The survey was first developed in English and then translated into French. The French translation was carried out by a French native speaker and bilingual OT and subsequently back translated by an English native speaker and bilingual OT. Language revisions were made as necessary until the two versions were considered equivalent.

### Survey Content

The survey comprised five sections: Participants’ Characteristics, DCD Diagnostic Process, Assessment Practices, Intervention Practices, and Conclusion. The first section gathered generic information regarding personal factors (gender, age, and highest degree of education achieved) and service characteristics (province or territory of employment, employment status and setting, clinical experience with children with DCD, and proportion of caseload of children with DCD), while the second section focused on the involvement of OTs in the DCD diagnostic process at the participant's institution. The Assessment Practices and Intervention Practices sections constituted the core of the survey and addressed approaches and modalities for each academic activity, for a maximum of six multiple-choice or short-ended questions per academic activity. The core sections focused on four specific academic activities, that is, handwriting (including legibility and speed, and keyboarding), mathematics (including numeration, algebra, geometry, measurements, data analysis and probability, mental computation, arithmetic and equations, problem-solving), writing (i.e., nonmotor domains of writing such as grammar, punctuation, sentence composition, organization of ideas, spelling), and reading (including reading fluency and comprehension). The concluding section was an open-ended question designed to gather general comments or feedback on the survey. The survey's structure followed a logical progression from general to specific questions, with branching that led to subquestions where appropriate. Most response options were closed-ended, although open-ended comments were allowed (optional) in specific instances to add to the richness of the data. Depending on the branching of their answers, participants completed between 26 and 72 questions over 17 web pages, for duration of up to 30 min.

### Data Analysis

Descriptive statistics were used to characterize the sample. Survey responses were quantified, coded, and tabulated in SPSS to pool the data together and perform the analyses (Corp., 2016). For continuous variables, results were presented using means and standard deviations, while categorical variables were computed in proportions and frequencies. Content analysis was conducted on open-ended questions to identify categories using inductive coding. The ranking question was analyzed by computing the average ranking and standard deviation for each possible response option and comparing each concurrent response option using independent t-tests. To better characterize the sample, Chi-square, and Fisher's exact tests were used to explore participant's personal factors and service characteristics. These same tests were used to investigate potential associations between personal factors or service characteristics and the type of academic activity assessed or treated. Following significant Chi-Square tests, post hoc analyses with Bonferroni correction for multiple comparisons were conducted to identify potential differences between OT practices across provinces.

## Results

### Participants

**Figure 1.**
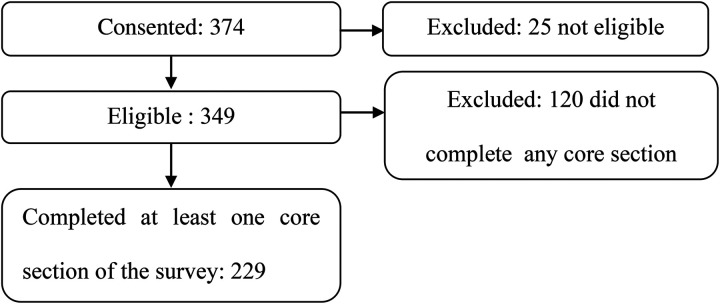
Participant flow diagram.

A total of 457 individuals viewed the survey and 374 consented to participate. Of these, 25 individuals (7%) were not eligible to participate after completing the eligibility questions and 120 (32%) did not complete the core sections of the survey (Assessment Practice and Intervention Practices sections) and were therefore excluded from the data analysis. The final sample included 229 participants, with three participants (1%) not completing the Intervention Practices section (Figure 1).

In all provinces, recruitment was facilitated through their respective OT professional agencies, except for British Columbia (BC), where consenting members were directly contacted by the study coordinator, which resulted in a larger participation in BC (Table 1) than in the other provinces. Ten participants (4%) indicated working as an OT in more than one province.

**Table 1.** Participant Personal Factors and Service Characteristics (*n* = 229).

			*n* (%)
Participant's personal factors	Gender	Female	170 (74)
		Male	53 (23)
		Prefer not to answer	6 (3)
	Age (years)	20–29	49 (21)
		30–39	109 (48)
		40–49	44 (19)
		50 or more	21 (9)
		Prefer not to answer	6 (3)
	Highest degree of education achieved	Bachelors	56 (24)
		Professional Masters	125 (55)
		Research masters	40 (17)
		Doctorate	8 (3)
Service characteristics	Province or territory of employment	British Columbia	91 (40)
		Quebec	62 (27)
		Ontario	28 (12)
		Newfoundland and Labrador	15 (7)
		Alberta	12 (5)
		Manitoba	10 (4)
		Northwest Territories	8 (3)
		New-Brunswick	5 (2)
		Nova Scotia	5 (2)
		Nunavut	3 (1)
		Saskatchewan	3 (1)
		Prince Edward Island	2 (1)
		Yukon	1 (0)
	Employment status	Employed	194 (85)
		Self-employed	35 (15)
	Employment setting	Community-based services	57 (25)
		Rehabilitation center	56 (24)
		Hospital	53 (23)
		Private clinic	36 (16)
		School board	27 (12)
	Clinical experience with children with DCD (years)	0–5	97 (42)
		6–10	79 (35)
		>11	53 (23)
	Proportion of caseload of children with DCD	>70%	38 (17)
		≈50%	73 (32)
		≈30%	86 (38)
		<10%	32 (14)

The majority of the participants were English-speaking (*n* = 175, 76%), self-identified as females (*n* = 170, 74%), in their 30s (*n* = 109, 48%), with a professional Masters of OT as highest degree of education (*n* = 125, 55%). Francophone participants were all from the province of Quebec, except 2 (96%). Due to the OT University programs nationwide transitioning from a bachelor degree to a professional master degree in the early 2000s, participants with a bachelor degree as highest degree of education were at least 40 years of age and more likely to have extensive experience working with children with DCD. Hence, age and experience were negatively associated with higher levels of education in OTs (χ^2^(12, *n* = 229) = 91.451, p < .001 and χ^2^(12, *n* = 229) = 60.825, p < .001). Years of experience working with children with DCD was not significantly associated with the proportion of children with DCD on the participant's caseloads (χ^2^(12) = 19.833, p = .070). Participants reported working on average 37±11 h per week as pediatric OTs (ranging from 4 to 70 h weekly, median = 36 h). A total of 111 participants (48%) worked with a clientele mainly composed of children with DCD (≥50%). With regard to the type of service delivered, 52% of participants (n = 118) indicated providing a combination of direct assessments, direct interventions, and indirect services, such as consultative services, providing coaching and training, and participating in advocacy activities, 34% (n = 78) indicated providing direct assessment and intervention, while only 1% (n = 2) indirect service only (Supplemental Figure 1). The detailed nature of the assessment and intervention practices is reported in subsequent sections.

### Survey

#### DCD Diagnostic Process

Sixty-seven percent of all participants (n = 153/229) were considered to have at least an advanced level of knowledge of the DSM-5's diagnostic criteria for DCD. A total of 81% of participants indicated that OTs at their institution were involved in the diagnostic process for DCD most of the time, and only five participants reported no involvement in this capacity. When asked about potential reasons for their partial or noninvolvement, the reasons included lack of awareness of the expertise of OTs among other health professionals (33%), limited access to OT or other multidisciplinary services (28%), systemic barriers (limited time or resources, long waitlists, not within their mandate) (19%) and poor knowledge of DCD (14%). Detailed answers regarding the DCD diagnostic process are available in Supplemental Table 1. Participants were asked to rank the professionals most involved (ranked first) in the diagnostic process of children with DCD in their practice, to least involved (ranked last). To the respondents’ knowledge, the most involved professional was the physician (including pediatrician and family doctor) with a median ranking of 1.5, closely followed by OTs with a median ranking of 2. Physicians and OTs were most often ranked as first for their diagnostic involvement. They were followed by neuropsychologists (median = 3), physiotherapists (median = 4), and psychologists (median = 4), all three of which ranked closely with no significant differences among them. Supplemental Table 2 provides detailed description of the involvement of professionals in the diagnostic process, including median, mode, and percentage of rankings.

#### Assessment Practices

All participants but three indicated that they directly assessed academic activities in children with DCD (n = 226/229, 99%). Handwriting was the academic activity most frequently assessed (96%), followed by writing (74%), mathematics (72%), and reading (66%) (Table 2). The most common reasons provided for not assessing a specific academic activity included mandate limitations (42%), lack of knowledge and/or training (20%), and lack of requests (8%).

**Table 2.** Domains of Academic Activities Addressed by OTs in School-Aged Children with DCD.

		Assessment (*n* = 229) *n* (%)	Intervention (*n* = 220) *n* (%)
Handwriting		220 (96)	187 (85)
	Handwriting speed	186 (81)	135 (61)
	Handwriting legibility	160 (70)	147 (67)
	Keyboarding	102 (45)	94 (43)
	All domains	83 (36)	71 (32)
Mathematics		164 (72)	149 (68)
	Numeration	78 (34)	75 (34)
	Arithmetic and equations	68 (30)	66 (30)
	Measurements	57 (25)	58 (26)
	Problem solving	53 (23)	47 (21)
	Algebra	50 (22)	45 (20)
	Geometry	45 (20)	49 (22)
	Mental computation	36 (16)	44 (20)
	Data analysis and probability	29 (13)	35 (16)
	All domains	12 (5)	5 (2)
Writing		170 (74)	144 (65)
	Sentence composition	107 (47)	87 (40)
	Punctuation	96 (42)	74 (34)
	Organization of ideas	88 (24)	79 (36)
	Grammar	71 (38)	52 (24)
	Spelling	62 (27)	41 (19)
	All domains	28 (12)	16 (7)
Reading		151 (66)	130 (59)
	Reading fluency	114 (50)	71 (32)
	Reading comprehension	92 (40)	104 (47)
	All domains	55 (24)	45 (20)

The components and modalities of assessments were not significantly different between each academic activity (Table 3). Assessments most often targeted activity performance (63%–85%) and personal factors (47%–60%), which were defined as individual intrinsic characteristics independent of the health condition and included motivation toward the activity, perceived self-esteem, or self-efficacy. Direct task observation (58%–65%) and interviews (50%–69%) were mostly used to assess academic activities. Usage of standardized assessments was reported in 39% of participants for handwriting, 7% for reading and writing, and 3% for mathematics. When questioned about the name of the standardized assessments used, participants identified 27 assessments for handwriting, six for writing, six for reading, and three for mathematics, although not all of these were standardized (Supplemental Table 3).

**Table 3.** Assessment Practices of OTs Toward School-Aged Children with DCD (*n* = 229).

		**Handwriting** ***n* (%)**	**Mathematics** ***n* (%)**	**Writing** ***n* (%)**	**Reading** ***n* (%)**
**Any type of assessment**		220 (96)	164 (72)	170 (74)	151 (66)
**Assessment components**	Activity performance	187 (85)	111 (68)	116 (68)	95 (63)
	Personal factors	132 (60)	77 (47)	87 (51)	72 (48)
	Body function and structure	133 (60)	60 (37)	65 (38)	55 (36)
	Environmental factors	115 (52)	67 (41)	55 (32)	49 (32)
**Assessment** **modalities**	Direct task observations	143 (65)	95 (58)	101 (59)	87 (58)
	Interview	151 (69)	84 (51)	91 (54)	75 (50)
	Questionnaires completed by parent, child, teacher, other professional or other	112 (51)	64 (39)	64 (38)	62 (41)
	Prior documentation	107 (49)	68 (41)	62 (36)	44 (29)
	Questionnaires completed by OT	66 (30)	47 (29)	51 (30)	43 (28)
	Formal assessments	85 (39)	5 (3)	12 (7)	10 (7)

#### Intervention Practices

Thirty-one of the 220 participants (14%) who filled out the Intervention Practices section did not provide intervention regarding academic activities to school-aged children with DCD, two of which specifically indicated not assessing these activities in children with DCD (1%). For the 189 who did, handwriting was the academic activity most often addressed by OTs (85%), followed by mathematics (79%), writing (77%), and reading (70%) (Table 2). The main reasons for not providing intervention for a specific academic activity were mandate limitations (62%), lack of requests to address this academic activity (14%), lack of knowledge and/or training from the OT (11%), and time constraints (3%).

To characterize their intervention practices, OTs detailed their practices with regard to direct intervention, which included remedial intervention and environmental or task modifications, and indirect intervention, which included consultative services, providing coaching and training, and participating in advocacy activities. A total of 78% of OTs reported providing direct intervention for handwriting difficulties, 60% for mathematics, 55% for writing, and 50% for reading. Fifty-one percent of OTs reported delivering indirect intervention, such as coaching and training, for handwriting difficulties, 30% for mathematics, 35% for writing, and 25% for reading, oriented mostly toward teachers and school personnel (88%–91%) and parents and caregivers (71%–85%). Among participants who directly provided intervention for academic activities, the CO-OP approach was the most frequently used for mathematics (70%), writing (62%), and reading (64%). For handwriting, motor learning approaches prevailed (87%). Across academic activities, the preferred modifications were adapted stationery, tools, and pencils (51%–80%). The detailed intervention modalities are presented in Table 4. As for referral practices, up to 94% of participants indicated referring children with academic difficulties to other professionals, primarily to speech language therapists in presence of handwriting (32%) or reading difficulties (53%), and to teaching specialists in presence of difficulties in mathematics (48%) or writing (51%) (Table 5).

**Table 4.** Intervention Practices of OTs for School-Aged Children with DCD (*n* = 220).

			Handwriting *n* (%)	Mathematics *n* (%)	Writing *n* (%)	Reading *n* (%)
Any type of intervention			**187 (85)**	**149 (68)**	**144 (65)**	**130 (59)**
	Direct services		**171 (78)**	**131 (60)**	**122 (55)**	**111 (50)**
		Environmental or task modifications	**142 (65)**	**107 (49)**	**101 (46)**	**88 (40)**
		Adapted stationery	113 (51)	71 (32)	66 (30)	45 (20)
		Adapted tools and pencils	111 (50)	69 (31)	64 (29)	45 (20)
		Visual cues and memory aids	110 (50)	70 (32)	61 (28)	39 (18)
		Technological aids	99 (45)	62 (28)	55 (25)	45 (20)
		Task presentation modifications	90 (41)	57 (26)	57 (26)	39 (18)
		Adapted furniture	90 (41)	38 (17)	42 (19)	28 (13)
		Time modifications	77 (35)	47 (21)	45 (20)	24 (11)
		Sensory tools	72 (33)	24 (11)	26 (12)	16 (7)
		Remedial intervention	**109 (50)**	**84 (38)**	**66 (30)**	**55 (25)**
		CO-OP	79 (36)	59 (27)	41 (19)	35 (16)
		Motor learning (skill acquisition and training)	95 (43)	51 (23)	39 (18)	30 (14)
		Cognitive approaches	62 (28)	51 (23)	40 (18)	24 (11)
		Behavioral approaches	36 (16)	34 (15)	22 (10)	17 (8)
		Biomechanical approaches	38 (17)	22 (10)	19 (9)	12 (5)
		Neurodevelopmental therapy	27 (12)	21 (10)	13 (6)	19 (9)
		Sensory integration therapy	36 (16)	15 (7)	12 (5)	12 (5)
	Indirect services		**113 (51)**	**66 (30)**	**78 (35)**	**55 (25)**
		To teachers and school personnel	99 (45)	58 (26)	71 (32)	48 (22)
		To parents and caregivers	96 (44)	50 (23)	61 (28)	39 (18)
		To school boards	32 (15)	19 (9)	24 (11)	11 (5)

*Note:* CO-OP = Cognitive Orientation to daily Occupational Performance. To facilitate data readability, the main category responses are in bold.

**Table 5.** Referrals in Presence of Academic Difficulties (*n* = 220).

		Handwriting *n* (%)	Mathematics *n* (%)	Writing *n* (%)	Reading *n* (%)
Referral to any profession		167 (76)	181 (82)	193 (88)	190 (86)
Profession referred to	Speech-language pathologists	71 (32)	50 (28)	92 (48)	101 (53)
	Teaching specialists	67 (30)	87 (48)	98 (51)	84 (44)
	Neuropsychologists	47 (21)	46 (25)	36 (19)	41 (22)
	Psychologists	44 (20)	41 (23)	37 (19)	41 (22)
	OTs	6 (3)	1 (1)	0 (0)	0 (0)
	Optometrists	1 (1)	1 (1)	1 (1)	5 (2)

#### Associations Between Personal Factors or Service Characteristics and Type of Academic Activities

Potential associations between personal or service characteristics and the type of academic activity assessed or treated were investigated to explore trends in OT practices. OTs those whose highest degree of education was a professional OT degree (Bachelor or Master's) were less likely to assess mathematics (only 68% of OTs whose highest degree was professional assessed mathematics vs 92% of OTs with a postprofessional degree, p ≤ .001) and reading (61% vs 89%, p ≤ .001), and to provide intervention for mathematics (64% vs 81%, p = .005), reading (51% vs 89%, p ≤ .001), and writing (59% vs 89%, p ≤ .001) than those with an additional postprofessional degree (e.g., research or secondary Master's degree, or doctorate) (α_adj_ = .008).

Additionally, OTs with a majority of children with DCD on their caseload (≥50%) were more likely to assess mathematics (90% of OTs with ≥50% of children with DCD on their caseload assessed mathematics versus 56% OTs with ≤30%, χ^2^(1) = 27.701, p < .001), reading (89% vs 46%, χ^2^(1) = 50.704, p < .001), and writing (86% vs 65%, χ^2^(1) = 8.737, p = .003) or to provide intervention for these activities (mathematics: 89% vs 49%, χ^2^(1) = 41.628, p < .001; reading: 87% vs 33%, χ^2^(1) = 73.720, p < .001; writing: 86% vs 46%, χ^2^(1) = 37.178, p < .001) than OTs with a smaller number of children with DCD in their caseload (≤30%) (α_adj_ = .008).

When comparing school-based OTs to those from other settings (i.e., community, rehabilitation centers, hospitals, and private practices all together), school-based OTs were found to assess significantly less often mathematics (only 44% of school-based OTs evaluated mathematics vs 76% of OTs from other settings, p = .001) or reading (41% vs 70%, p = .004) and provided intervention significantly less often for handwriting (63% vs 88%, p = .002), mathematics (33% vs 73%, p < .001), reading (22% vs 64%, p < .001), and writing (37% vs 70%, p = .002) (α_adj_ = .01). The main reasons listed by school-based OTs for not assessing or providing intervention for these activities were mandate limitations (68%), lack of knowledge and/or training from the OT (14%) or time constraints (11%).

Significant provincial differences were found in assessment practices for reading (χ^2^(12) = 35.254, p < .001) and writing (χ^2^(12) = 54.230, p < .001), as well as intervention practices for mathematics (χ^2^(12) = 26.805, p = .008), reading (χ^2^(12) = 38.956, p < .001), and writing (χ^2^(12) = 37.224, p < .001). Post hoc analyses revealed that participants from the province of Quebec less frequently assessed reading (only 41% of OTs from Quebec assessed reading vs 80% of OTs from BC; χ^2^(1) = 13.107, p = .0003) and writing (44% vs 71%; χ^2^(1) = 33.835, p < .0001) than those from British Columbia. Additionally, participants from Quebec less frequently assessed reading (41% vs 100%; χ^2^(1) = 12.984, p = .0003) and less frequently provided intervention for reading (38% vs 100%; χ^2^(1) = 14.511, p = .0001), and writing (40% vs 100%; χ^2^(1) = 13.389, p = .0002) than those from Newfoundland & Labrador. All other pairwise comparisons were not significant after Bonferroni correction (α_adj_ = 0.0006).

## Discussion

In addition to primary dysfunction in motor domains, children with DCD more frequently experience challenges in academic activities than typically developing peers (Dionne et al., 2022) and need intervention to support their participation and performance in these activities. As an initial step to ensure that Canadian children with DCD receive the appropriate services, we conducted the first extensive pan-Canadian survey on the assessment and intervention practices of OTs working with this population in diverse settings.

The survey revealed that almost all participants indicated assessing or providing intervention on at least one academic activity (99%). This widespread involvement aligns with current guidelines on the management of DCD, which recommend addressing all the activity-based needs of children with DCD, not just motor-based difficulties (Blank et al., 2019; Camden et al., 2015). The OTs sampled reported typically assessing performance in all academic activities, but only up to 60% included personal factors, such as motivation toward the activity or perceived self-esteem, in their assessment, despite the significant impact these may have on academic performance (Khanna et al., 2016). A total of 39% of OTs reported using formal assessments to evaluate handwriting (39%), as opposed to 7% or less for mathematics, reading, and writing, logically aligning with the predominance of challenges in motor-based activities among children with DCD (Dhall, 2016). Listed formal assessments included the McMaster Handwriting Assessment Protocol (Pollock et al., 2009) for handwriting, the Developmental Eye Movement Test (Richman, 1987) for reading, and the KeyMath Diagnostic Assessment (Conolly, 2007) for mathematics. In terms of intervention practices, CO-OP and motor learning approaches were preferred, consistent with evidence-based recommendations for intervention in children with DCD (Preston et al., 2017; Smits-Engelsman et al., 2013), whereas approaches specific to learning disabilities, such as direct training and hands-on activities, were not mentioned (Gifford & Rockliffe, 2012; Nelson & Powell, 2018).

The survey revealed that for each academic activity, at least half of OTs reported providing interventions, yet they provided interventions directly and indirectly more frequently for handwriting than for any other academic activity. This could be expected for reading and writing, given that in children with DCD, difficulties with these activities are much less frequently reported than for handwriting (Blank et al., 2012; Dionne et al., 2022; Tseng et al., 2007). However, difficulties in mathematics are prevalent, affecting up to 90% of children with DCD (Dionne et al., 2022), yet only 72% of respondents assessed mathematical performance, and even fewer provided intervention for this activity. Given that mathematical challenges are often associated with language difficulties (Chow et al., 2021) and cognitive impairments (Schwenck et al., 2015), children facing these issues may already be receiving services from neuropsychologists or special educators for instance. Interestingly, OTs with a large proportion of children with DCD in their caseload tended to assess and provide intervention for mathematics more often than those with fewer cases, possibly suggesting that OTs more specialized with children with DCD have heightened awareness of their common mathematical difficulties. Raising awareness in the broader OT community about the common mathematical challenges faced by children with DCD and the role of OTs in addressing these difficulties may be a simple yet effective solution to promote positive practice changes, encouraging OTs to either address mathematical difficulties or refer to other professionals.

Raising awareness can be effectively achieved through information dissemination initiatives as is currently recommended for physicians, teachers, and caregivers, but directed toward OTs (Camden et al., 2015; Wilson et al., 2013; Withers et al., 2017). For instance, these initiatives could involve organizing professional development seminars or workshops or providing informational capsules in relevant OT electronic newsletters. OTs with a postprofessional degree assessed and provided intervention more frequently for mathematics, reading, and writing than those with a professional OT degree. Given that OTs with additional degrees tend to be younger and will eventually represent an increasingly significant portion of the OT workforce, a higher proportion of OTs incorporating all academic activities in their practices can be anticipated. Whether this reflects a change in OT curricula remains to be determined. Nevertheless, this trend, alongside ongoing information dissemination initiatives, could support better OT management of children with DCD and more holistic services for children with DCD.

Most surveyed participants indicated that OTs were generally involved in the diagnostic process for DCD, though not as frequently as physicians (and psychologists in some jurisdictions), who have diagnostic privileges. While the reported 30% of physicians knowledgeable about DCD in 2013 has likely increased over the following decade (Wilson et al., 2013), the fact that two-thirds of OTs self-reported having an expert or advanced level of knowledge regarding the DCD diagnostic criteria from the DSM-5 validates the importance of involving OTs as experts in the DCD diagnostic process, as recommended by the Canadian Association of Occupational Therapy (OT) (CAOT, 2018). The main reason for not involving OTs was a lack of awareness of their expertise among other health professionals, suggesting that advocacy efforts remain pertinent to sustain and potentially increase the frequency of OT involvement in diagnosing DCD.

Regarding the practices of school-based OTs for academic activities, these OTs provided intervention services less frequently for academic activities than those working in other settings, reporting mandate restrictions and time limitations as the most frequent barriers to providing this service. Although this may highlight a potential gap in school-based OT services, this could also indicate that these children may be receiving support from other sources within the school system, such as resource or special education teachers. However, parents of children with DCD have previously reported a lack of school-based therapy support (Klein et al., 2024), suggesting that the resources currently available do not meet the needs of children with DCD. Collaboration between OTs and educational professionals would ensure bridging the medical and educational systems, as knowledge of health conditions is essential to provide inclusive and targeted instruction to support academic activities (Florian & Black-Hawkins, 2011), and children with DCD have multidimensional needs to reach success (Nabors et al., 2008; Wilson et al., 2013). Canadian OTs are recognized for being very knowledgeable of DCD and their unique challenges (Karkling et al., 2017); however, parents have reported a lack of school-based therapy support in the provinces of British Columbia (Klein et al., 2024) and Ontario (Missiuna, Moll et al., 2006b), suggesting that the resources currently available do not meet the needs of children with DCD. Due to the nature of their training and the specificity of the profession, OTs are uniquely positioned to assess and support children's global functioning. Consequently, OTs should be integral members of the multidisciplinary team supporting children with DCD and academic difficulties.

Although further research is needed to identify the specific barriers that limit school-based OT interventions on academic activities in Canada, it is likely that improving the efficiency of OT services using consultative models such as “Partnering for Change”, a collaborative approach to service delivery that integrates OTs directly into school and community settings, could help OTs allocate more resources toward academic activities while supporting as many children as possible and fostering sustainable change (Campbell et al., 2016; Missiuna, Gaines et al., 2006a; Missiuna et al., 2012; Miyahara et al., 2009; Reid et al., 2006; Rens & Joosten, 2014; Wehrmann et al., 2006). However, the survey reported that Canadian OTs favored direct intervention (50%–78%) as opposed to consultation or indirect intervention (25%–51%). Therefore, future studies should look at exploring how to effectively implement consultative or indirect models of OT service delivery are essential in Canada. Implementing meaningful changes toward indirect service delivery will have a positive impact on OT services and will support OTs in addressing all academic activities for children with DCD.

## Limitations

Certain limitations in this study must be acknowledged. Firstly, using a convenience sample may have introduced a potential sampling bias, as the survey likely reached OTs who are involved or interested in academic activities, consequently providing different answers from nonparticipants. In this study, participants from British Columbia were overrepresented, whereas females, francophones, and school-based OTs were fewer than the proportion reported in the pool of Canadian OTs (Canada, 2023). Furthermore, provincial differences in education and healthcare service organization may explain the variability in practices across Canada. To recognize these disparities, we explored associations between various personal and service characteristics and presented the results by province, gender, language, education, experience, and setting where applicable. Secondly, the self-report nature of the survey may have introduced a respondent social desirability bias, with OTs potentially aligning their answers with best practices instead of reflecting their actual practices, as well as a recall bias, since OTs were reporting on their practices over time. These biases were likely consistent in each participant's answers, therefore skewing answers toward more desirable answers, but still allowing for individual differences to be apparent. Finally, the survey's length may have affected participant participation in the later sections of the survey. However, there were no significant differences between the personal factors or service characteristics of participants who partially completed the survey and those who completed it in full, indicating reliability in partial completions. Overall, the study's findings offer a cross-sectional take on OTs current practices across Canada, and therefore must be interpreted with caution when compared to future studies.

## Conclusion

Canadian OTs frequently assess and provide evidence-based interventions for academic activities in children with DCD. However, their practices remain predominantly focused on handwriting, with comparatively less emphasis on mathematics, writing, and reading, despite the high frequency of these difficulties in this population. Additionally, raising awareness among OTs regarding the significance of the various academic difficulties of children with DCD and their potential role in improving academic outcomes for children with DCD is warranted, especially among school-based OTs, and may broaden the focus of their interventions.

## Key Messages

- Most Canadian Occupational Therapists reported assessing and intervening on academic activities in children with Developmental Coordination Disorder.
- Canadian Occupational Therapists more frequently provided direct intervention for children with Developmental Coordination Disorder compared to consultative services or indirect intervention.
- Future initiatives should focus on continuing to raise Occupational Therapists’ awareness of the academic difficulties of children with Developmental Coordination Disorder and their role in supporting academic success.

## Supplemental Material

sj-docx-1-cjo-10.1177_00084174251359768 - Supplemental material for Occupational Therapy for Children with DCD and Academic Difficulties: A Pan-Canadian Survey Supplemental material, sj-docx-1-cjo-10.1177_00084174251359768 for Occupational Therapy for Children with DCD and Academic Difficulties: A Pan-Canadian Survey by Eliane Dionne, Annette Majnemer, Miriam H. Beauchamp and Marie Brossard-Racine in Canadian Journal of Occupational Therapy

sj-docx-2-cjo-10.1177_00084174251359768 - Supplemental material for Occupational Therapy for Children with DCD and Academic Difficulties: A Pan-Canadian Survey Supplemental material, sj-docx-2-cjo-10.1177_00084174251359768 for Occupational Therapy for Children with DCD and Academic Difficulties: A Pan-Canadian Survey by Eliane Dionne, Annette Majnemer, Miriam H. Beauchamp and Marie Brossard-Racine in Canadian Journal of Occupational Therapy

sj-docx-3-cjo-10.1177_00084174251359768 - Supplemental material for Occupational Therapy for Children with DCD and Academic Difficulties: A Pan-Canadian Survey Supplemental material, sj-docx-3-cjo-10.1177_00084174251359768 for Occupational Therapy for Children with DCD and Academic Difficulties: A Pan-Canadian Survey by Eliane Dionne, Annette Majnemer, Miriam H. Beauchamp and Marie Brossard-Racine in Canadian Journal of Occupational Therapy

sj-docx-4-cjo-10.1177_00084174251359768 - Supplemental material for Occupational Therapy for Children with DCD and Academic Difficulties: A Pan-Canadian Survey Supplemental material, sj-docx-4-cjo-10.1177_00084174251359768 for Occupational Therapy for Children with DCD and Academic Difficulties: A Pan-Canadian Survey by Eliane Dionne, Annette Majnemer, Miriam H. Beauchamp and Marie Brossard-Racine in Canadian Journal of Occupational Therapy

sj-docx-5-cjo-10.1177_00084174251359768 - Supplemental material for Occupational Therapy for Children with DCD and Academic Difficulties: A Pan-Canadian Survey Supplemental material, sj-docx-5-cjo-10.1177_00084174251359768 for Occupational Therapy for Children with DCD and Academic Difficulties: A Pan-Canadian Survey by Eliane Dionne, Annette Majnemer, Miriam H. Beauchamp and Marie Brossard-Racine in Canadian Journal of Occupational Therapy
