# Supplementary material for: Occupational Therapy for Children With DCD and Academic Difficulties: A Pan-Canadian Survey
Source: Can J Occup Ther. 2025 Jul 30;93(3):329–40. doi: 10.1177/00084174251359768 (PMC13400826; doi:10.1177/00084174251359768)
Supplement: sj-docx-1-cjo-10.1177_00084174251359768 - Supplemental material for Occupational Therapy for Children with DCD and Academic Difficulties: A Pan-Canadian Survey [file sj-docx-1-cjo-10.1177_00084174251359768.docx]

#### Supplemental Table 1. DCD diagnostic knowledge and involvement (n=229)

|  | | n (%) |
| --- | --- | --- |
| Self-perceived level of knowledge of DCD diagnostic criteria from DSM-5 | Expert | 33 (14) |
|  | Advanced | 120 (52) |
|  | Intermediate | 64 (28) |
|  | Beginner | 11 (5) |
|  | No knowledge | 1 (0) |
| Frequency of OT involvement in diagnostic process | Always | 100 (44) |
|  | Most of the time | 85 (37) |
|  | Sometimes | 39 (17) |
|  | Never | 5 (2) |
