# Supplementary material for: Occupational Therapy for Children With DCD and Academic Difficulties: A Pan-Canadian Survey
Source: Can J Occup Ther. 2025 Jul 30;93(3):329–40. doi: 10.1177/00084174251359768 (PMC13400826; doi:10.1177/00084174251359768)
Supplement: sj-docx-2-cjo-10.1177_00084174251359768 - Supplemental material for Occupational Therapy for Children with DCD and Academic Difficulties: A Pan-Canadian Survey [file sj-docx-2-cjo-10.1177_00084174251359768.docx]

#### Supplemental Table 2. Ranking distribution by professionals most involved in the diagnostic process for DCD according to participants (n=229)

|  | Mean (SD) | Median | Mode | 1^st^ rank frequency n(%) | 2^nd^ rank frequency n(%) | 3^rd^ rank frequency n(%) |
| --- | --- | --- | --- | --- | --- | --- |
| Physician, pediatrician or family doctor | 2.4(2.2) | 1.5 | 1 | 93(41) | 37(16) | 17(7) |
| OT | 3.2(3.0) | 2 | 1 | 67(29) | 30(13) | 22(10) |
| Physiotherapist | 4.7(2.7) | 4 | 3 | 7(3) | 20(9) | 34(15) |
| Neuropsychologist | 4.8(3.2) | 3 | 3 | 4(2) | 29(13) | 38(17) |
| Psychologist | 4.9(3.3) | 4 | 4 | 16(7) | 14(6) | 13(6) |
| Special educator | 5.8(3.6) | 5 | 3 | 10(4) | 11(5) | 14(6) |
| Neurologist | 6.0(3.4) | 6 | 2 | 4(2) | 13(6) | 3(1) |
| Teacher | 6.6(4.2) | 5 | 5 | 4(2) | 13(6) | 10(4) |
| Psychiatrist | 6.6(3.8) | 6 | 4 | 4(2) | 7(3) | 10(4) |
| Speech language pathologist | 7.2(3.7) | 7 | 5 | 7(3) | 1(0) | 6(3) |
| Nurse | 7.4(3.8) | 8 | 11 | 4(2) | 9(4) | 2(1) |
| Kinesiologist | 7.6(3.6) | 8 | 10 | 3(1) | 7(3) | 0(0) |
| Case manager | 8.0(4.0) | 8 | 5 | 1(0) | 3(1) | 3(1) |
| Social worker | 8.1(3.9) | 8 | 13 | 5(2) | 2(1) | 2(1) |
