# Supplementary material for: Occupational Therapy for Children With DCD and Academic Difficulties: A Pan-Canadian Survey
Source: Can J Occup Ther. 2025 Jul 30;93(3):329–40. doi: 10.1177/00084174251359768 (PMC13400826; doi:10.1177/00084174251359768)
Supplement: sj-docx-3-cjo-10.1177_00084174251359768 - Supplemental material for Occupational Therapy for Children with DCD and Academic Difficulties: A Pan-Canadian Survey [file sj-docx-3-cjo-10.1177_00084174251359768.docx]

#### Supplemental Table 3. Assessment tools listed by academic activity

|  | Assessment tool | Abbreviation | Construct assessed | Frequency (n) | Standardized or not | Reference |
| --- | --- | --- | --- | --- | --- | --- |
| Handwriting | McMaster Handwriting Assessment Protocol* | MHA | Handwriting | 67 | NS | (Pollock et al., 2009) |
|  | Beery-Buktenica Developmental Test of Visual-Motor Integration* | Beery-VMI | Visual-motor integration | 36 | S | (Beery & Beery, 2010) |
|  | Movement Assessment Battery for Children, 2^nd^ edition | M-ABC2 | Motor skills | 16 | S | (Brown & Lalor, 2009) |
|  | Bruininks-Oseretsky Test of Motor Proficiency, 2^nd^ edition | BOT2 | Motor skills | 10 | S | (Bruininks, 2005) |
|  | Evaluation Tool of Children’s Handwriting* | ETCH | Handwriting | 8 | NS | (Amundson, 1995) |
|  | Test of Visual Perceptual Skills | TVPS | Visual perception | 5 | S | (Martin, 2017) |
|  | ABC Boum Handwriting Assessment Procedure | - | Handwriting | 4 | NS |  |
|  | Detailed Assessment of Speed of Handwriting* | DASH | Handwriting speed | 3 | S | (Barnett et al., 2007) |
|  | Developmental Coordination Disorder Questionnaire | DCDQ | Motor coordination | 3 | S | (Wilson et al., 2007) |
|  | Handwriting without Tears’ Screener of Handwriting Proficiency | - | Handwriting Proficiency | 3 | NS | (Olsen, 2003) |
|  | Miller Function & Participation Scales | M-FUN | Functional motor skills | 3 | S | (Miller, 2006) |
|  | *Échelle d’évaluation rapide de l’écriture chez l’enfant* | BHK | Handwriting | 2 | S | (Charles & Michel, 1986) |
|  | Developmental Test of Visual Perception* | DTVP | Visual perception and visual-motor integration | 2 | S | (Frostig et al., 2013) |
|  | Handwriting Interactive Assessment Tool* | HIAT | Handwriting performance | 2 | NS | (*Handwriting Interactive Assessment Tool*, N.D.) |
|  | *Test de Manipulation des outils scolaires* | Man.OS | Handwriting | 2 | NS | (Lefévère, 2010) |
|  | Perceived Efficacy and Goal Setting System | PEGS | Self-efficacy and goal setting | 2 | NS | (Missiuna et al., 2004) |
|  | Brock String Assessment | - | Oculomotor skills | 1 | NS | (Brock, 1950) |
|  | Developmental Coordination Disorder Checklist | DCD checklist | Motor coordination | 1 | NS | (Wilson, 2007) |
|  | Daily Questionnaire for Developmental Coordination Disorder | DCDDaily-Q | Motor coordination | 1 | S | (Schoemaker & van Netten, 2012) |
|  | *Échelle Victor-Doré* | - | Motor development | 1 | S | (Victor-Dore, 2016) |
|  | *Épreuve de Vitesse d’Écriture de* Dauphin Penmanship Test | EVEDP | Handwriting speed | 1 | S | (Alexandre, 1981) |
|  | Minnesota Handwriting assessment | MHA | Handwriting | 1 | S | (Reisman, 1999) |
|  | Schoodles School Fine Motor Assessment | Schoodles | Fine motor skills | 1 | NS | (Frank, 2019) |
|  | Sensory Profile | SP | Sensory processing | 1 | S | (Dunn, 2014) |
|  | Sensory Processing Measure | SPM | Sensory processing | 1 | S | (Henry et al., 2007) |
|  | This is how I write: A Child’s Self-Assessment of Handwriting | - | Handwriting | 1 | NS | (Goldstand et al., 2013) |
|  | Vestibular/Ocular Motor Screening | VOMS | Vestibular and oculomotor functions | 1 | S | (*Vestibular/Ocular Motor Screening*, N.D.) |
| Writing | McMaster Handwriting Assessment Protocol* | MHA | Handwriting | 9 | NS | (Pollock et al., 2009) |
|  | Test of Written Language | TOWL | Written language | 2 | S | (Hammill & Larsen, 2009) |
|  | Detailed Assessment of Speed of Handwriting* | DASH | Handwriting speed | 1 | S | (Barnett et al., 2007) |
|  | Evaluation Tool of Children’s Handwriting* | ETCH | Handwriting | 1 | NS | (Amundson, 1995) |
|  | Handwriting Interactive Assessment Tool* | HIAT | Handwriting performance | 1 | NS | (*Handwriting Interactive Assessment Tool*, N.D.) |
|  | Wilson Assessment of Decoding and Encoding* | WADE | Reading and spelling | 1 | NS | (Wilson, 1996) |
| Reading | Developmental Eye Movement Test | DEM | Oculomotor skills | 3 | S | (Richman, 1987) |
|  | Northeastern State University College of Optometry Oculomotor Test | NSUCO | Oculomotor skills | 3 | S | (Optometry, N.D.) |
|  | McMaster Handwriting Assessment Protocol* | MHA | Handwriting | 2 | NS | (Pollock et al., 2009) |
|  | Cognitive Abilities Test | CogAT | Cognitive abilities | 1 | S | (Lohman, 2011) |
|  | Peabody Picture Vocabulary Test | PPVT | Receptive language | 1 | S | (Dunn, 2020) |
|  | Wilson Assessment of Decoding and Encoding | WADE | Reading and spelling | 1 | NS | (Wilson, 1996) |
| Mathematics | KeyMath Diagnostic Assessment | KeyMath | Mathematical skills | 3 | S | (Conolly, 2007) |
|  | Developmental Test of Visual Perception* | DTVP | Visual perception and visual-motor integration | 2 | S | (Frostig et al., 2013) |
|  | Beery-Buktenica Developmental Test of Visual-Motor Integration* | Beery-VMI | Visual-motor integration | 1 | S | (Beery & Beery, 2010) |

Legend: NS, non-standardized; S, standardized.

*Assessment used for more than one academic activity.

Note: Words in *italics* are the official names of the assessments in French that have not been formally translated to English.

## References

Alexandre, A. (1981). *Dauphin Penmanship Test*.

Amundson, S. J. (1995). *Evaluation Tool of Children's Handwriting*. Therapy Skills Builders.

Barnett, A., Henderson, S., Scheib, B., & Schulz, J. (2007). *Detailed Assessment of Speed of Handwriting*. Pearson.

Beery, K. E., & Beery, N. A. (2010). *Beery-Buktenica Developmental Test of Visual-Motor Integration, 6th ed.* (6th edition ed.). Pearson.

Brock, F. W. (1950). *Brock String Assessment*.

Brown, T., & Lalor, A. (2009). The Movement Assessment Battery for Children-Second Edition (MABC-2): A Review and Critique. *Physical & Occupational Therapy In Pediatrics*, *29*(1), 86-103.

Bruininks, R. H. B., B.D. (2005). *Bruininks-Oseretsky Test of Motor Proficiency* (2nd ed.). Pearson.

Charles, M.-M., & Michel, J.-M. (1986). *Échelle d’évaluation rapide de l’écriture chez l’enfant*. Éditions du Centre de Psychologie Appliquée (CPA).

Conolly, A. J. (2007). *KeyMath Diagnostic Assessment, 3rd Canadian edition* (3 ed.). Pearson Assessment.

Dunn, D. M. D., L. M. (2020). *Peabody picture vocabulary test*. Pearson.

Dunn, W. (2014). *Sensory Profile 2*. Pearson.

Frank, M. F., M. (2019). *Schoodles School Fine Motor Assessment, 4th edition*. Schoodles.

Frostig, M., Hammill, D. D., Pearson, N. A., & Voress, J. L. (2013). *Developmental Test of Visual Perception 3rd edition*. Pro-Ed, Inc.

Goldstand, S., Gavir, D., Cermak, S. A., & Bissel, J. (2013). *This is how I write: A Child’s Self-Assessment of Handwriting*. Therapro, Inc.

Hammill, D. D., & Larsen, S. C. (2009). *Test of Written Language*. PRO-ED.

*Handwriting Interactive Assessment Tool*. (N.D.).

Henry, D. P., D., Kuhaneck, H., Glennon, T., & Reynolds, S. (2007). *Sensory Processing Measure*. Western Psychological Services.

Lefévère, G. (2010). *Test de Manipulation des outils scolaires, Man.Os*.

Lohman, D. F. (2011). *Cognitive Abilities Test Form 7 (CogAT)*. Riverside Insights.

Martin, N. A. (2017). *Test of Visual Perceptual Skills* (4th edition ed.). Academic Therapy Publications.

Miller, L. J. (2006). *Miller Function & Participation Scales*. Pearson Clinical Assessment.

Missiuna, C., Pollock, N., & Law, M. (2004). Perceived Efficacy and Goal Setting System (PEGS). In. San Antonio, TX: Psychological Corporation.

Olsen, J. Z. (2003). *Handwriting Without Tears 5th edition*.

Optometry, N. S. U. C. o. (N.D.). *Oculomotor Test*.

Pollock, N., Missiuna, C., & Hoare, P. (2009). *McMaster Handwriting Assessment Protocol*. McMaster University.

Reisman, J. (1999). *Minnesota Handwriting Assessment*. Harcourt Assessment, Inc.

Richman, J. G., D. (1987). *Developmental Eye Movement Test*. Bernell Corporation.

Schoemaker, M. M., & van Netten, J. M.-v. d. L., B. W. . (2012). *Daily Questionnaire for Developmental Coordination Disorder*. ZGT Hospital.

*Vestibular/Ocular Motor Screening*. (N.D.).

Victor-Dore, F. (2016). *Échelle Victor-Doré*.

Wilson, B. A. (1996). *Wilson Assessment of Decoding and Encoding*. Wilson Language Training Corporation.

Wilson, B. C., S. G. (2007). *Developmental Coordination Disorder Checklist*. Alberta Children's Hospital

Wilson, B. N., Kaplan, B. J., Crawford, S. G., Roberts, G., & Dewey, D. (2007). *Developmental Coordination Disorder Questionnaire*. Alberta Children's Hospital.
