# Supplementary material for: Occupational Therapy for Children With DCD and Academic Difficulties: A Pan-Canadian Survey
Source: Can J Occup Ther. 2025 Jul 30;93(3):329–40. doi: 10.1177/00084174251359768 (PMC13400826; doi:10.1177/00084174251359768)
Supplement: sj-docx-4-cjo-10.1177_00084174251359768 - Supplemental material for Occupational Therapy for Children with DCD and Academic Difficulties: A Pan-Canadian Survey [file sj-docx-4-cjo-10.1177_00084174251359768.docx]

#### Supplemental Figure 1. Venn diagram of the type of services offered by the participants.

n=118 (52%)

n=22 (10%)

n=78 (34%)

n=1 (0%)
