# Supplementary material for: Occupational Therapy for Children With DCD and Academic Difficulties: A Pan-Canadian Survey
Source: Can J Occup Ther. 2025 Jul 30;93(3):329–40. doi: 10.1177/00084174251359768 (PMC13400826; doi:10.1177/00084174251359768)
Supplement: sj-docx-5-cjo-10.1177_00084174251359768 - Supplemental material for Occupational Therapy for Children with DCD and Academic Difficulties: A Pan-Canadian Survey [file sj-docx-5-cjo-10.1177_00084174251359768.docx]

#### Supplemental Materials. Survey distributed to potential participants

1. Are you currently a licensed OT in Canada?
   - Yes
   - No
2. Do you have at least one year of clinical experience with a paediatric clientele?
   - Yes
   - No
3. In the last year, did you perform at least one assessment or intervention session with a school-aged client with DCD?
   - Yes
   - No
4. How do you currently self-identify?
   - Male
   - Female
   - Other: ______________
   - Prefer not to answer
5. What is your age?
   - 20-29 years
   - 30-39 years
   - 40-49 years
   - 50 or more
   - Prefer not to disclose.
6. Select the highest degree of education you obtained:
   - Bachelors
   - Masters (professional)
   - Masters (research)
   - Doctorate
   - Other, please specify: ____________________
7. In which province or territory do you work ~~in~~ as a pediatric OT?
   - Alberta
   - British Columbia
   - Manitoba
   - New-Brunswick
   - Newfoundland and Labrador
   - Northwest Territories
   - Nova Scotia
   - Nunavut
   - Ontario
   - Prince Edward Island
   - Quebec
   - Saskatchewan
   - Yukon
8. How many hours per week do you work as a pediatric OT?
   - Number of hours: ____________________
9. What is your employment status where you most often work with children with DCD (if you are currently on leave, please answer according to your latest status)?
   - Employed
   - Self-employed
   - Other, please specify: ____________________
10. Which setting best describes where you most often work with children with DCD?
    - Rehabilitation centre
    - Community-based services (e.g., CLSC/community health centres, community services)
    - Hospital setting (i.e., inpatient or outpatient)
    - Private practice
    - School board
    - Other, please specify: ____________________
11. How many years of clinical experience do you have as an OT working with children with DCD?
    - < 1 year
    - 1 to 5 years
    - 6 to 10 years
    - 11 to 20 years
    - 21 years and more
12. Select the roles that best describe your mandate as an OT providing services to children with DCD. *Check all that apply.
    - Consultation or indirect services (e.g., consultative services with school boards or teachers, providing training to teachers and/or parents, lobbying)
    - Direct assessment of the child
    - Direct intervention (e.g., 1:1 or group)
    - Other, please specify: ____________________
13. Approximately what percentage of your clientele are children with DCD?
    - Most of my clientele has DCD (i.e., ≥ 70% of my clientele)
    - Approximately half of my clientele has DCD (i.e., ≈50% of my clientele)
    - Some of my clientele has DCD (i.e., ≈30% of my clientele)
    - Very few of my clientele has DCD (i.e., $\leq$10% of my clientele)

For your information, here are the official DCD criteria from the DSM-V:

A. The acquisition and execution of coordinated motor skills is substantially below that expected given the individual’s chronological age and opportunity for skill learning and use. Difficulties are manifested as clumsiness (e.g., dropping or bumping into objects) as well as slowness and inaccuracy of performance of motor skills (e.g., catching an object, using scissors or cutlery, handwriting, riding a bike, or participating in sports).

B. The motor skills deficit in Criterion A significantly and persistently interferes with activities of daily living appropriate to chronological age (e.g., self-care and self-maintenance) and impacts academic/school productivity, prevocational and vocational activities, leisure, and play.

C. Onset of symptoms is in the early developmental period.

D. The motor skills deficits are not better explained by intellectual disability (Intellectual developmental disorder) or visual impairment and are not attributable to a neurological condition affecting movement (e.g., cerebral palsy, muscular dystrophy, degenerative disorder).

1. How would you describe your level of knowledge regarding the diagnostic **criteria** for DCD from the DSM-V?
   - Expert level
   - Advanced level
   - Intermediate level
   - Beginner level
   - No knowledge
2. To your knowledge, how frequently are OTs involved in the diagnostic **process** for the children with DCD that you see in your practice?
   - Always
   - Most of the time
   - Sometimes
   - Never
3. Why do you think OTs in your practice are not always involved in the diagnostic **process** of children with DCD? (*question only appears if previous answer is “Most of the time”, “Sometimes” or “Never”)
4. Rank in order of frequency in terms of the involvement of all the professionals who, to your knowledge, are involved in the diagnostic **process** of the children with DCD you see in your practice. Exclude any professionals who are not involved in the diagnostic process.
   #1 would be most involved, then #2 next most involved and so forth (involved based on number of hours of service provided), leaving out those not involved at all.
   - Physician, pediatrician or family doctor
   - Occupational Therapist
   - Physiotherapist
   - Speech language pathologist
   - Psychologist
   - Neuropsychologist
   - Psychiatrist
   - Kinesiologist
   - Special educators
   - Social worker
   - Nurse
   - Case manager
   - Neurologist
   - Other:

This survey focuses on **academic activities**, which are the scholarly competencies (not school subjects) that children typically acquire in school (e.g., literacy and numeracy, which include learning to write, read, spell, and count). This excludes activities that pertain to daily living skills (e.g., walking, dressing, eating).

1. Do you assess elementary school-aged children with DCD?*
   - Yes
   - No. (*skip to Treatment Practices section)
     - Why don’t you assess elementary school-aged children with DCD? ______

*The following four questions appears four times, one for each of the following academic activities:*

- 1. Handwriting (and/or keyboarding)
  2. Writing (i.e., non-motor aspects of writing such as grammar, punctuation, sentence composition, organization of ideas, spelling)
  3. Reading
  4. Mathematics (e.g., numeration, arithmetic, problem solving)

1. Do you assess _a,b,c,d___ ? If so, how often?
   - Most of the time (i.e., ≥ 70% of the time)
   - Many times (i.e., ≈ 50% of the time)
   - Sometimes (i.e., ≈ 30% of the time)
   - Rarely (i.e., ≤ 10% of the time)
   - No, I do not assess this activity. (*Skip next two questions)
     - Why don’t you assess this activity? _________________
2. Which aspects of _a,b,c,d___ do you assess in elementary school-aged children with DCD?
   - Handwriting
     - Handwriting legibility
     - Handwriting speed
     - Keyboarding
   - Writing skills
     - Grammar
     - Punctuation
     - Sentence composition
     - Organization of ideas
     - Spelling
   - Reading
     - Reading comprehension
     - Reading fluency
   - Mathematics
     - Numeration
     - Algebra
     - Geometry
     - Measurements (e.g.: time, money, distance)
     - Data analysis and probability
     - Mental computation
     - Arithmetic and equations (Additions, subtractions, multiplications and divisions)
     - Problem-solving
3. When you assess _a,b,c,d____, what do you assess specifically? *Check all that apply.
   - Activity performance (e.g., speed, positioning, reading proficiency, neatness of work, use of tools)
     - Please specify which components of activity performance you assess: ____
   - Personal factors (e.g., motivation towards the activity, perceived self-esteem or self-efficacy)
     - Please specify which personal factors you assess: ____
   - Environmental factors (e.g., accessibility restrictions, teaching methods)
     - Please specify which environmental factors you assess: ____
   - Underlying components or deficits (e.g., attention span, visuomotor integration, memory)
     - Please specify which underlying components you assess: ____
   - Other
     - Please specify what other components you assess: _____
4. How do you assess _a,b,c,d___? *Check all that apply.
   - Interview (e.g., structured or semi-structured, with parents, teachers, other professionals or other)
   - Questionnaires completed by OT
   - Questionnaires completed by parent, child, teacher, other professional or other
   - Direct Task observations (e.g., during schoolwork)
   - Prior documentation (e.g., report cards, professional reports, school samples)
   - Standardized assessments
     - Which standardized assessments do you use to assess_a,b,c,d___ in school-aged elementary children with DCD : _________
   - Other; please specify: ______________
5. If you have any additional comments regarding your assessment practices of academic activities in school-aged children with DCD, please write them here.

This survey focuses on **academic activities**, which are the scholarly competencies (not school subjects) that children typically acquire in school (e.g., literacy and numeracy, which include learning to write, read, spell, count). This excludes activities that pertain to daily living skills (e.g., walking, dressing, eating).

1. Do you treat (directly, or indirectly by consultation) elementary school-aged children with DCD?*
   - Yes
   - No. (*Skip to conclusion)
     - Why don’t you treat elementary school-aged children with DCD? ______

Ask the following 2 questions for each following academic activity:

- 1. Handwriting (and/or keyboarding)
  2. Writing (i.e., non-motor aspects of writing such as grammar, punctuation, sentence composition, organization of ideas, spelling)
  3. Reading
  4. Mathematics (e.g., numeration, geometry, mental computation, problem solving)

1. Do you provide treatment for children who experience __a, b, c, d__ difficulties? *Check all that apply.
   - Yes; I use remediation approaches. *Check all that apply.
     - Cognitive Orientation to daily Occupational Performance (CO-OP)
     - Motor learning (skill acquisition and training)
     - Cognitive approaches
     - Behavioral approaches
     - Biomechanical approaches
     - Neurodevelopmental therapy
     - Sensory integration therapy
     - Other: ______________
   - Yes; I use environmental or task modifications. *Check all that apply.
     - Technological aids (e.g.: computer, tablets, cell phone and/or applications)
     - Adapted tools and pencils (e.g.: adapted or specific pencil, erasers, protractors)
     - Adapted stationery (e.g.: specialized paper, graph paper, legal-size paper)
     - Visual cues and memory aids (e.g.: visual cues to sequence or organize the task or sequencing memory aids)
     - Task presentation modifications (e.g.: one exercise per page, one-sided paper, size of writing on paper, verbal explanations)
     - Adapted furniture (e.g.: desk, chair, cushion)
     - Time modifications (e.g.: sequencing the task, additional time)
     - Sensory tools (e.g.: fidget tools, noise-cancelling headphones)
   - Yes; I provide education, coaching and/or consultation services. *Check all that apply.
     - For school boards
     - For teachers and school personnel
     - For parents and caregivers
     - Other, please specify: _________
   - No.
     - Why don’t you intervene on this activity? (*Skip next question for this activity)__________
   - Other; please specify: ________________
2. Which aspects of _a, b, c, d___ do you intervene on ?
   - Handwriting
     - Handwriting legibility
     - Handwriting speed
     - Keyboarding
   - Writing skills
     - Grammar
     - Punctuation
     - Sentence composition
     - Organization of ideas
     - Spelling
   - Reading
     - Reading comprehension
     - Reading fluency
   - Mathematics
     - Numeration
     - Algebra
     - Geometry
     - Measurements (e.g.: time, money, distance)
     - Data analysis and probability
     - Mental computation
     - Arithmetic and equations (Additions, subtractions, multiplications and divisions)
     - Problem solving
3. Do you refer children with _a, b, c, d___ difficulties elsewhere?
   - Psychologists
   - Neuropsychologists
   - Speech language pathologists (SLPs)à
   - Teaching specialists
   - Other
     - To whom do you refer elementary school-aged children with DCD who experience _a, b, c, d___ difficulties? ______
   - No; I don’t typically refer these children to other professionals or specialists (I.e., for any reason, including not part of your mandate or not necessary to refer).

If you have any additional comments regarding your treatment practices for school-aged children with DCD, please write them here.

1. Do you have any comments regarding this survey?

This completes the series of questions of this survey. We wish to thank you for participating in this survey and providing your responses.
